# Fluorescence-Based Ion Transport Assays Using Proteoliposomes

**DOI:** 10.21769/BioProtoc.5679

**Published:** 2026-04-20

**Authors:** Karthik Ramanadane, Elena F. Lehmann, Cristina Manatschal

**Affiliations:** 1 Department of Biochemistry, University of Zurich, Zurich, Switzerland; 2 Structural Biology Center, Fondazione Human Technopole, Milan, Italy

**Keywords:** Fluorescence-based in vitro transport assay, Metal ion transporters, Reconstituted proteoliposomes, Calcein, Fura-2, Magnesium Green, ACMA

## Abstract

Divalent metal ion transporters are conserved across all domains of life and play essential roles in diverse processes such as manganese acquisition during nutritional immunity in bacteria and iron homeostasis in higher eukaryotes [1–3]. Traditional techniques, such as electrophysiological assays, are often unsuitable due to the slow kinetics of many membrane transporters, electroneutral nature of certain transporter types, and the influence of other proteins with similar activity. To overcome these limitations and to investigate both the activity and ion selectivity of transporters, also including those normally expressed intracellularly, we have developed a fluorescence-based transport assay using purified proteins. This in vitro assay uses encapsulated fluorophores to monitor the movement of divalent metal ions (e.g., Mn^2+^, Ca^2+^, Mg^2+^) or protons across liposomal membranes reconstituted with purified transporter proteins. This approach provides detailed functional insight that complements structural and cellular data.

Key features

• Enables detection of real-time transport activity through precise timing of reagent addition and controlled generation of membrane potential.

• Compatible with a wide range of divalent metal ions and ionophores, allowing adaptation to various transporter types.

• Applicable to transporters that are naturally expressed in intracellular compartments, but requires a purified protein sample.

• Allows detailed analysis of transporter function in a defined lipid environment and testing effects of binders and compounds.

## Graphical overview


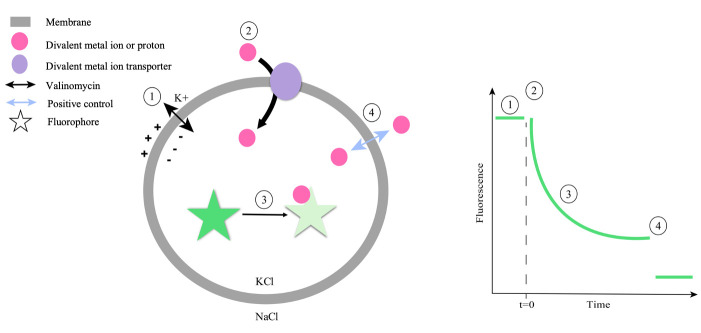


**Schematic overview of setup and time flow of a fluorescence-based ion transport assay to detect proton-coupled metal ion symport.** Upon addition of the potassium ionophore valinomycin, an outwardly directed potassium gradient establishes a negative membrane potential **(1)**. In the presence of this negative membrane potential, the reconstituted transporter mediates fast electrogenic symport of divalent metal ions and protons. In the absence of this additional driving force, the transport proceeds with slower kinetics **(2)**. Transport activity is monitored by recording changes in the fluorescence of an encapsulated fluorophore that responds to changes in the internal ion concentration, such as Calcein for Mn^2+^ ions, ACMA for H^+^, Fura-2 for Ca^2+^, or Magnesium Green for Mg^2+^. **(3)**. As a positive control, an appropriate ionophore is added to equilibrate the ions across the membrane, resulting in maximal fluorescence change **(4)**. The scheme reflects the expected signal change upon detection of Mn^2+^ transport using Calcein as a fluorophore and Calcimycin as a positive control.

## Background

This protocol allows the characterization of transport kinetics and substrate specificity of purified transporters. These types of assays are also well suited for structure-function analyses of wild-type and mutant proteins as well as for testing inhibitors and other regulatory factors [4–15]. Traditionally, electrophysiological approaches such as voltage-clamp recordings [16–19] or radioactive tracer [20,21] assays have been used to study metal ion transport. While valuable, these methods can be influenced by other membrane proteins with similar activity, limited by technical complexity, low temporal resolution, or incompatibility with certain transporter types. Fluorescent metal ion indicators have also been applied in live-cell studies [22], but controlling the ionic environment and membrane potential in such systems remains challenging. The fluorescence-based in vitro transport assay presented here overcomes some of these limitations by reconstituting purified transporters into artificial liposomes loaded with fluorophores. This artificial environment allows monitoring ion transport under controlled conditions, including defined ionic composition, lipid environment, and membrane potential. A defined membrane potential can be established by applying a potassium concentration gradient in combination with the potassium ionophore valinomycin, for example, to enable efficient transport or to investigate the stoichiometry of electrogenic transporters [23–25]. One important limitation of the protocol is that transporter orientation within liposomes cannot be precisely controlled, leading to a mix of outside-out and inside-out populations. Although some membrane proteins may insert preferentially in one direction under certain conditions [26–30], this was not the case for divalent metal ion transporters investigated in our studies [12]. Transporter orientation needs to be taken into account when interpreting kinetic data with respect to the used membrane potential and when exploring the effects of inhibitors or regulatory factors, as access to the relevant binding site depends on the transporter’s orientation. This protocol specifically focuses on assays to investigate the transport of divalent ions and protons as good ionophores for direct detection exist. To investigate the transport of monovalent ions, assays have to be modified in order to detect transport activity indirectly; these were described previously, for instance, for SLC26 transporters and Orai channels [31,32].

## Materials and reagents

**Biological materials**

1. Membrane transporter reconstituted into liposomes

2. Liposomes devoid of proteins (negative control)

**Reagents**

1. Chloroform (Fluka, catalog number: 25690)

2. Diethyl ether (Sigma, catalog number: 296082)

3. Calcein (Thermo Fisher Scientific, catalog number: C481)

4. Fura-2 (Thermo Fischer Scientific, catalog number: F1200)

5. Magnesium Green (Thermo Fischer Scientific, catalog number: M3733)

6. Valinomycin (Thermo Fischer Scientific, catalog number: V1644)

7. Calcimycin (Thermo Fischer Scientific, catalog number: V1644)

8. Ionomycin (Thermo Fischer Scientific, catalog number: I24222)

9. 9-amino-6-chloro-2-methoxyacridine (ACMA) (Thermo Fischer Scientific, catalog number: A1324)

10. Carbonyl cyanide m-chlorophenyl hydrazone (CCCP) (Thermo Fischer Scientific, catalog number: C2759)

11. Sodium chloride (NaCl) (Sigma, catalog number: 71380)

12. Potassium chloride (KCl) (Sigma, catalog number: 746436)

13. HEPES (Sigma, catalog number: H3375)

14. Manganese chloride (MnCl_2_) (Fluka, catalog number: 31422)

15. Magnesium chloride (MgCl_2_) (Fluka, catalog number: 63065)

16. Calcium chloride (CaCl_2_) (Fluka, catalog number: 223506)

**Solutions**

1. Storage buffer (see Recipes)

2. Buffer IN (see Recipes)

3. Buffer IN with fluorophore (see Recipes)

4. Buffer OUT (see Recipes)

5. Valinomycin, Calcimycin, Ionomycin, and CCCP stock solutions (see Recipes)

6. ACMA stock solution (see Recipes)

7. Metal ion stock solutions (see Recipes)

**Recipes**

**1. Storage buffer**

**Table:** 

Reagent	Final concentration	Quantity or volume
HEPES pH 7.5, 1 M	20 mM	1 mL
KCl, 2 M	100 mM	2.5 mL
Deionized water	n/a	To 50 mL

**2. Buffer IN**

**Table:** 

Reagent	Final concentration	Quantity or volume
HEPES pH 7.0, 1 M	20 mM	1 mL
KCl, 2 M	100 mM	2.5 mL
Deionized water	n/a	To 50 mL

**3. Buffer IN with fluorophore**

**Table:** 

Reagent	Final concentration	Quantity or volume
HEPES pH 7.0, 1 M	20 mM	0.1 mL
KCl, 2 M	100 mM	0.25 mL
Fluorophore		Respective quantity to reach 250 μM Calcein, 100 μM Fura-2, or 400 μM Magnesium Green
Deionized water	n/a	To 5 mL

For Calcein, it is possible to prepare a stock solution of higher concentration that can be stored for several weeks at 4 °C. Due to reduced stability and high costs for Fura-2 and Magnesium Green, it is advisable to use freshly prepared solutions and smaller volumes. Calcein, Fura-2, and Magnesium Green are light sensitive.

**4. Buffer OUT**

**Table:** 

Reagent	Final concentration	Quantity or volume
HEPES pH 7.0, 1 M	20 mM	1 mL
NaCl, 5M	100 mM	1 mL
Deionized water	n/a	To 50 mL

**5. Valinomycin, Calcimycin, Ionomycin, and CCCP stock solutions**

**Table:** 

Reagent	Final concentration	Quantity or volume
Ionophore solution 500 μM (see below)	10 μM	200 μL
100% EtOH		9.8 mL
Total		10 mL

A 5 mM stock solution in 100% EtOH can be stored at -80 °C for long term (several months).

The 10 μM stock solution in 100% EtOH used in the experiment can be stored at -20 °C for several weeks.

Preparation of the 500 μM stock solution: dilute the 5 mM stock 1:10 with 100% EtOH to reach a concentration of 500 μM.

**6. ACMA stock solution**

**Table:** 

Reagent	Final concentration	Quantity or volume
ACMA solution 10 mM in 80% EtOH	1 mM	10 μL
20% EtOH		90 μL
Total		100 μL

A 10 mM stock solution in 80% EtOH can be stored at 4 °C for long term (several months). ACMA is not completely soluble in this mixture; therefore, mix shortly before use.

The 1 mM stock in 20% EtOH used in the experiment is soluble and can be stored at 4 °C for several months.

**7. Metal ion stock solutions**

**Table:** 

Reagent	Final concentration	Quantity or volume
MnCl_2_·4H_2_O (MW = 197.91 g/mL)	0.5 M	0.49 g
Deionized water	n/a	5 mL

**Table:** 

Reagent	Final concentration	Quantity or volume
MgCl_2_·6H_2_O (MW = 203.30 g/mol)	0.5 M	0.51 g
Deionized water	n/a	5 mL

**Table:** 

Reagent	Final concentration	Quantity or volume
CaCl_2_·2H_2_O (MW = 147.01 g/mol)	0.5 M	0.37 g
Deionized water	n/a	5 mL

These stock solutions are diluted to reach the desired concentrations needed in the experiments. For each experiment, freshly prepare metal ion stock solutions.

**Laboratory supplies**

1. Biobeads SM-2 adsorbents (Bio-Rad Laboratories, catalog number: 152-3920)

2. Microfuge tube polypropylene (Beckman Coulter, catalog number: 357448)

3. 96-well black walled microplates (Thermo Fischer Scientific, catalog number: M33089)

4. FIOLAX test glass tubes with beaded rim 12 mm × 100 mm (Duran Group, catalog number: 261101105)

## Equipment

1. Optima MAX-XP ultracentrifuge or related (Beckman Coulter, catalog number: 393315)

2. Rotor TLA 100.3 (Beckman Coulter, catalog number: 349490)

3. Diameter Delrin tube adapter 11 mm (Beckman Coulter, catalog number: 355919)

4. Avestin Extruder kit (Sigma, catalog number: Z373400)

5. Polycarbonate filters 400 nm (Sigma, catalog number: Z373435)

6. Tecan Infinite, Spark, or related (Tecan)

7. Bath sonicator model G112SPIT with power supply G112SPIG with transformer 2422-530-05415K (Laboratory Supplies Co. Inc, Hicksville, N.Y., and Filec, Netherlands)

## Procedure

As the reconstitution of proteins into liposomes is crucial for the transport assays and includes many delicate steps, we describe the procedure in **Section A**. Further details can be found in the work published by Geertsma et al. [28], which is the foundation of the protocol described below. Subsequently, unilamellar vesicles needed for the transport assays can be prepared using different techniques depending on the experimental objective. For metal ion transport assays, extrusion has proven to be the most suitable method, as it produces liposomes with a uniform and defined size, which is important for reproducibility and quantitative analysis (**Section B**). In contrast, for proton transport measurements, sensitivity is a limiting factor. The sensitivity of pH-sensitive fluorescence signals depends on the internal volume of the liposomes. Smaller vesicles enable more rapid and detectable pH changes upon proton flux. Therefore, sonication was found to be the preferred method for generating small unilamellar vesicles in this context (**Section C**). The protocol for recording the transport activity is finally described in **Section D**.

**A. Preparation of proteoliposomes**

*Note: As an example, the following steps describe the protocol used to generate proteoliposomes made of POPE:POPG (3:1) used in the study of the NRAMP-related Mg^2+^ transporter EleNRMT [14].*

1. Pool the desired quantity of lipids into a round-bottom glass flask. Pool 400 mg of lipids under the hood in a round-bottom glass flask. Wash the lipid vials with chloroform and pool in the same glass flask.

*Note: As an indication, 400 mg corresponds to 16 mL in chloroform, and 4 additional mL of chloroform were used, leading to 20 mL of solubilized lipids in a round-bottom glass flask of 500 mL capacity (11 cm in diameter, 18 cm height).*

2. Evaporate the chloroform with a fine stream of nitrogen by gently and uniformly rotating the flask until all the chloroform has dried and the lipids form a thin film within the flask.

*Note: To avoid any splashes, the stream should not be too strong. We recommend a stream that can be felt slightly when wearing nitrile gloves. Use bigger flasks when preparing bigger lipid batches, as the larger flask volume leads to a larger surface and thinner lipid film.*

3. Wash the lipids with diethyl ether (20 mL for 400 mg of lipids) and repeat the manual drying procedure until the lipids are dry. Continue drying with nitrogen for an additional 20–30 min under the hood.

4. Exchange the nitrogen–air mixture within the flask with argon. For this step, we recommend the use of a desiccator.

5. Dissolve lipids in 10 mL of storage buffer by sonication to a final concentration of 40 mg/mL. Each sonication cycle consists of 15 s sonication and 45 s on ice. Repeat until the lipids are fully solubilized into the storage buffer.

*Note: As each sonicator has its own settings, we recommend a sonication power that is high enough to solubilize the lipids but does not lead to splashing.*

6. Freeze-thaw the lipid mixture with liquid nitrogen at least 3 times and extrude the lipids at least 11 times using a 400 nm filter. Once extruded, keep the lipids at room temperature (>22 °C) and proceed with reconstitution.

**Pause points:** Lipids can be stored for up to one year at -80 °C after sonication, after freeze-thaw cycles, or after extrusion. Be aware that if the lipids are frozen after extrusion, the operator will need to repeat the extrusion before proceeding to the reconstitution.

7. Make each reconstitution sample with 5 mL of lipids diluted at 4 mg/mL with storage buffer. All the following steps are done at room temperature.

8. Proceed to liposome destabilization by adding 10% Triton X-100 and measuring the R_sat_ 540 nm. For a reconstitution of 5 mL at 4 mg/mL, one addition of Triton X-100 represents 10 μL. When R_sat_ is reached and the measurement starts to decrease, proceed to four more Triton X-100 additions by still waiting for the 30 s equilibration between each addition. The liposomes are now destabilized and ready for protein incorporation.

*Note: We use a small stirrer and wait 30 s after each addition to reach equilibrium before measurement.*

9. Add your protein of interest (POI) purified in detergent dropwise to the liposomes. Depending on the activity of your POI, we recommend testing different protein/lipid ratios (1:20, 1:50, 1:100, 1:200) and different lipid compositions.

*Note: Minimize air space between the solution and the cap of the tube, as this may lead to inactivation of proteins prior to insertion into lipid vesicles. The protein volume should not exceed 10% of the volume of liposomes.*

**Critical:** The proper mandatory negative control is the purification buffer with detergent devoid of protein. The negative control is particularly important as the quality of reconstitution and tightness of the proteoliposomes greatly depend on the detergent used for protein purification and its removal during the reconstitution process.

10. Incubate at room temperature for 15 min with gentle stirring.

11. Add 200 mg of biobeads (per 5 mL reconstitution) and incubate for 30 min at room temperature with gentle stirring. Again, add 200 mg of biobeads and incubate for 1 h at 4 °C with gentle stirring. Finally, add another 200 mg of biobeads and incubate overnight at 4 °C with gentle stirring.

**Critical:** The amount of biobeads added corresponds to biobeads without buffer after equilibration, following the manufacturer’s instructions. Biobeads are added to a Petri dish and incubated with methanol, water, and finally storage buffer for 5 min each. At each step, the equilibration solution is removed completely by using a Pipetboy and a 10 mL serological pipette. The Petri dish is slightly tilted, and the pipette tip is strongly pushed against the bottom edge of the Petri dish, thus allowing for complete buffer removal.

12. The next day, add another 200 mg of biobeads and incubate for 2 h at 4 °C.

13. Remove the biobeads using a gravity flow column with a filter and pellet the liposomes by centrifugation at 240,000× *g* for 40 min at 22 °C (we generally use an MLA55 or Ti70 rotor). Resuspend the proteoliposomes with storage buffer to a final concentration of 40 mg/mL and freeze/store in liquid nitrogen.

**Critical:** Proteoliposomes can lose activity over time due to freeze/thaw cycles. We thus recommend aliquoting the proteoliposomes, flash freezing them in liquid nitrogen, and storing them at -80 °C. A homogenous suspension can be reached by extruding the proteoliposomes before aliquoting and freezing.

**B. Preparation of liposomes for metal ion transport assay by extrusion**

1. Add the respective amount of proteoliposomes to 400 μL of buffer IN with fluorophore in a 2 mL Eppendorf tube.

*Note: An advisable amount of proteoliposomes to use for one series of experiments for one day is 1 mg of liposomes. The amount of proteoliposomes used refers to the amount of lipids at a certain protein-to-lipid ratio (w/w).*

2. Freeze the mixture in liquid nitrogen and thaw it again at room temperature. Repeat three times. Make sure that liposomes are warmed up to room temperature after thawing, especially before extrusion. We advise wiping off the condensate outside the tube and assessing that they are at room temperature by finger-touch after each freeze/thaw cycle. If the tube is tilted in a slight horizontal direction for freezing, the thawing process will be faster and easier to evaluate.

*Note: In case of light-sensitive fluorophores, protect from light using aluminum foil. The described fluorophores (Calcein, Fura-2, and Magnesium Green) are light sensitive.*

3. Assemble the extruder and wash the extruder first with buffer IN, then with buffer IN with fluorophore (250 μM Calcein, 100 μM Fura-2, or 400 μM Magnesium Green). Add the proteoliposomes to one side and extrude 9–11 times using a 400 nm filter. Rinse the extruder with 500 μL of buffer IN with fluorophore by extruding a couple of times. Add the rinsing solution to the liposomes.

**Figure 1.**
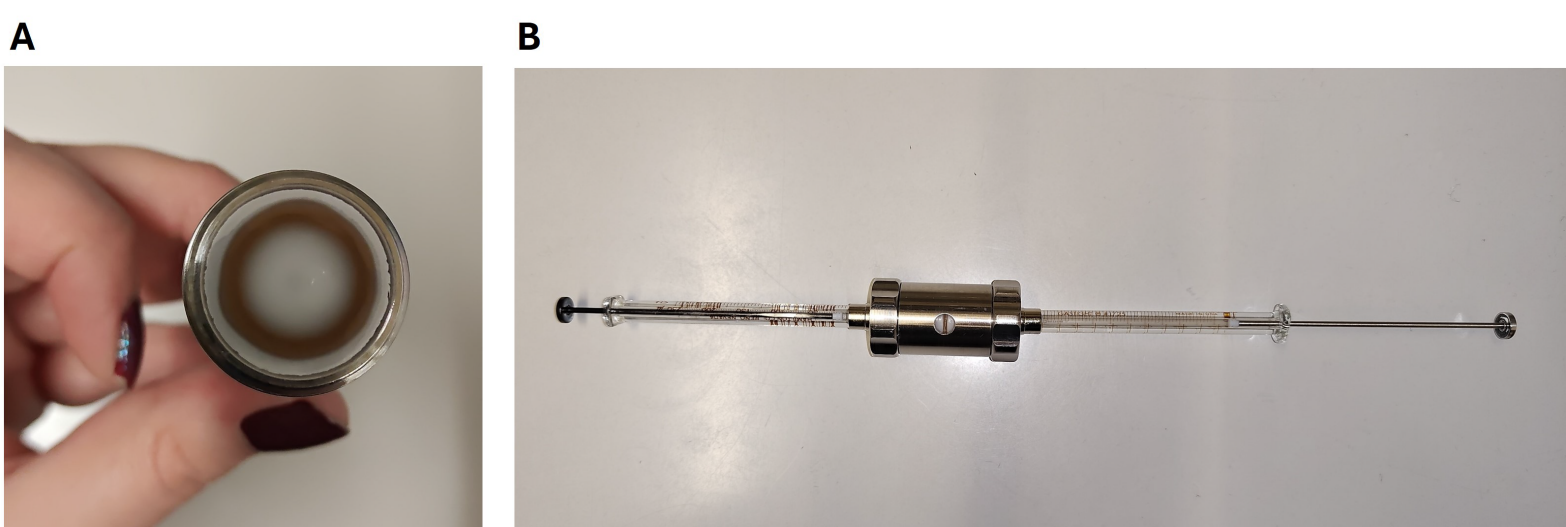
Assembly of the extruder. (A) It is important to place the filter without folds and air bubbles. (B) The extruder contains two syringes, one of which is used to load the lipids and the other one to collect the lipids.

**Figure 2.**
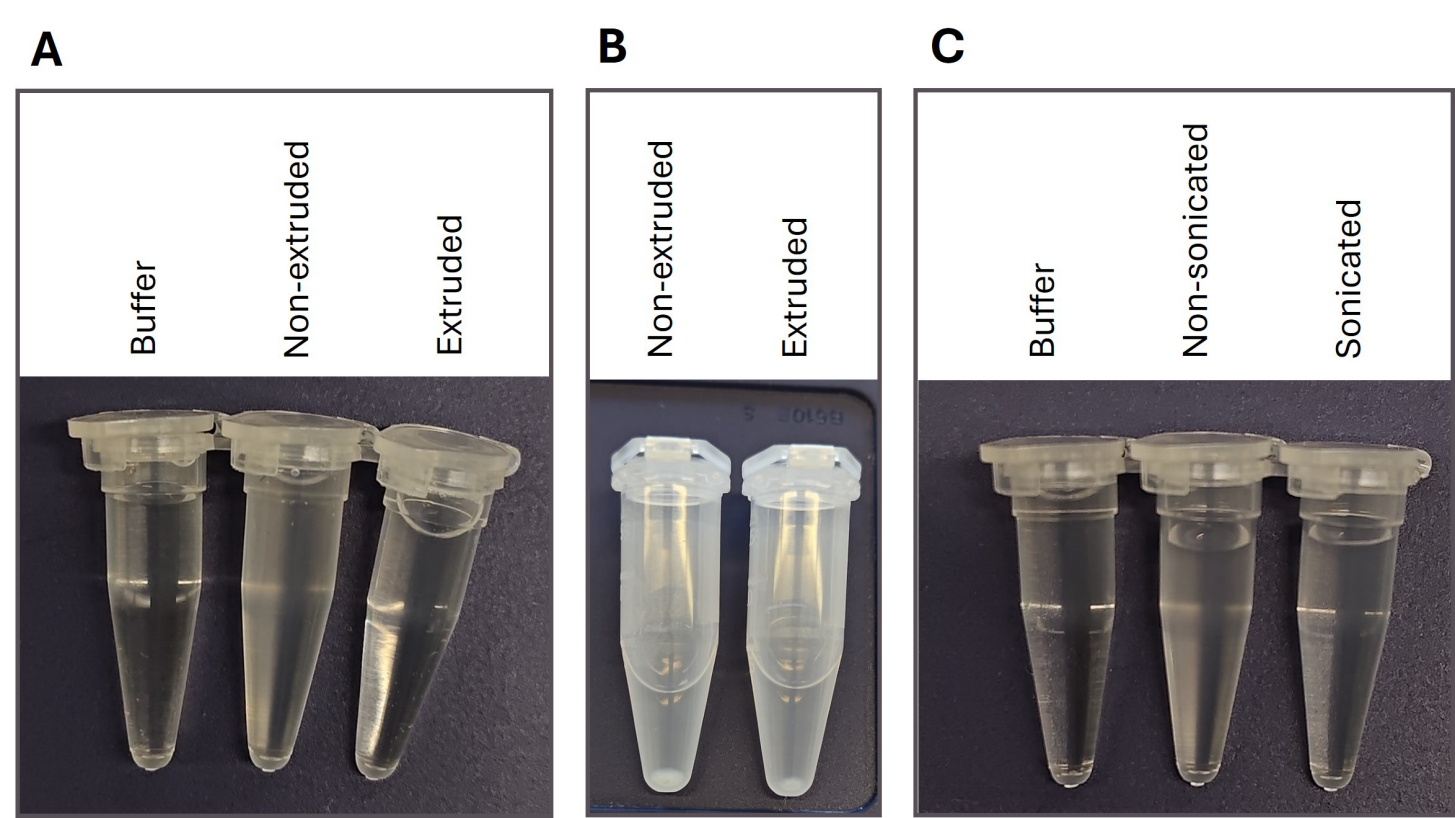
Appearance of proteoliposomes. (A) The difference in transparency between non-extruded and extruded proteoliposomes is illustrated and compared to a buffer solution. (B) After centrifugation, a soft pellet at the bottom of the tube becomes visible. With extruded proteoliposomes, the pellet is more transparent. (C) The difference in transparency between non-sonicated and sonicated proteoliposomes is illustrated and compared to a buffer solution.

*Note: When assembling the extruder, ensure that the membrane filter lies flat and does not form folds. Assemble the lower part first, place the filter onto the support after moistening it with a small amount of buffer, make sure to remove air bubbles, and then assemble the upper part (Figure 1). Extrude an uneven number of times to collect the extruded liposomes from the side that was NOT used to load the extruder. Make sure that the extruder is properly washed, dried, and at room temperature before each use. Extruded liposomes appear transparent, especially when held against a dark background (Figure 2A). With extruded lipids, the volume markings on the extruder syringe on the opposite side of the suspension are easier to read.*

**Critical:** After the extrusion step, always keep the liposomes above the phase transition temperature of the used lipid mixture.

4. Pellet the liposomes by centrifugation at 170,000× *g* for 25 min at 22 °C using a TLA 100.3 rotor and polypropylene tubes. The 400 μL extruded proteoliposomes and the 500 μL buffer used for the wash step result in approximately 900 μL per tube.

*Note: The specified centrifugation force corresponds to the maximum value set on the centrifuge. Due to the time required to reach the target g-force, the effective time at this force is shorter. We do not recommend using a swinging-bucket rotor, as this makes it more difficult to locate the transparent pellet. The pellet at the bottom of the tube is slightly more transparent for extruded as compared to non-extruded proteoliposomes (Figure 2B).*

**Critical:** Make sure that the rotor and the tube adapters are equilibrated to room temperature beforehand to avoid a temperature drop below the phase transition temperature. Often, rotors are kept at 4 °C and need several hours to equilibrate to room temperature. It is therefore advisable to place the rotor at room temperature the evening before the planned experiment.

5. Wash step: Carefully remove the supernatant and gently resuspend the pellet containing the liposomes in 800 μL of buffer IN. Pellet the liposomes by centrifugation as described above. We advise cutting the first millimeters of the P1000 tip extremity, thus allowing for gentle resuspension of the pellet without disrupting liposomes.

6. Repeat the wash step, for a total of two wash steps.

7. To reach a final concentration of 0.25 mg/mL, carefully resuspend the pellet in a total volume of 40 μL of buffer IN. This is the liposome stock solution.

**C. Preparation of liposomes for proton transport assay by sonication**

1. Prepare a proteoliposome stock of 15 mg/mL in 5 mM HEPES, pH 7.0, 100 mM KCl, 50 μM ACMA. Depending on the composition of the reconstituted proteoliposomes, this can be reached in different ways. If the proteoliposome stock has sufficiently high concentration and the buffer composition is sufficiently low, this final composition can be reached by simple dilution. Alternatively, it can be reached by diluting into larger volumes of 5 mM HEPES pH 7.0, 100 mM KCl, and subsequent wash steps as described in steps A5–7. In this latter case, ACMA should be added in the last resuspension step.

*Note: The required buffer capacity may vary depending on the experimental goal and should be optimized accordingly. For the study of metal ion transporters in the Dutzler laboratory, HEPES buffer concentrations of 5–10 mM have proven to be adequate.*

2. To generate small unilamellar vesicles, sonicate the proteoliposome solution until it appears optically clear.

*Notes:*

*1. Optical clarity is easier to assess when the solution is placed in a glass tube with sufficient volume. It is therefore recommended to use a total volume of 50 μL and the glass tubes listed in the laboratory supply section. The sonication intensity can be adjusted by changing the position of the tube within the sonication bath, as energy distribution is not uniform and depends on bath geometry and water level. These parameters should be taken into account for optimization of the sonication setup. For our setup, the sonication bath was filled to approximately 2 cm below the rim and set to 80 V, and liposomes were sonicated for 30–60 s with short breaks.*

*2. Sonicated lipids appear transparent, especially when held against a dark background (Figure 2C).*

**Critical:** After the sonication step, always keep the liposomes above the phase transition temperature for the lipid mixture used.

3. Collect all liquid to the bottom by brief centrifugation at room temperature, as after sonication, droplets may adhere to the inner wall of the tube. This is the liposome stock solution.

**D. Recording of transport activity by change of fluorescence signal**

1. For each measurement series, up to 8 different conditions can be tested in parallel. To prepare, dilute the liposome stock solution about 100 times by adding 8 μL to 830 μL of Buffer OUT. For each experimental condition, place 100 μL of this dilution in a well of a 96-black walled microplate.

2. Use a plate reader such as the Tecan Infinite or Spark to record fluorescence. Set the device to measure as continuously as possible. For Tecan Infinite M100, we recorded fluorescence every 4 s for a total of up to 360 cycles (24 min). Fluorescence settings vary depending on the indicator:

a. Mn^2+^ detection with Calcein: use λ_ex_ = 492 nm; λ_em_ = 518 nm.

b. Ca^2+^ detection with Fura-2: use the ratio between Ca^2+^ bound and unbound state.

i. Ca^2+^-bound Fura-2: λ_ex_ = 340 nm; λ_em_ = 510 nm.

ii. Ca^2+^-unbound Fura-2: λ_ex_ = 380 nm; λ_em_ = 510 nm.

c. Mg^2+^ detection with Magnesium Green: λ_ex_ = 506 nm; λ_em_ = 531 nm.

d. Detection of pH change with ACMA: λ_ex_ = 412 nm; λ_em_ = 482 nm.

3. Perform the following steps during measurement:

a. Baseline recording: Begin by recording 60–90 cycles (approximately 4–6 min) to establish a stable baseline fluorescence.

b. Initiate membrane potential: Add 1 μL of valinomycin stock solution to each 100 μL sample to generate a negative membrane potential. Continue measuring for another 20–30 cycles (approximately 1.5–2 min).

c. Start transport: Add 1 μL of the respective metal ion stock solution (e.g., Mn^2+^, Ca^2+^, or Mg^2+^) to initiate transport activity. Monitor for 75–270 additional cycles (approximately 5–18 min) depending on your experimental goal.

d. Apply positive control: Add 1 μL of the respective ionophore to validate the maximum possible fluorescence signal (Calcimycin for Mn^2+^ and Mg^2+^, Ionomycin for Ca^2+^, and CCCP for protons). Record fluorescence for another 10–20 cycles (approximately 1–1.5 min) after ionophore addition.

**Critical:** It is important to be fast in order to record as much as possible of the initial transport activity. Therefore, it is advisable to use a programmable multichannel pipette. Aliquot the ionophore stock solutions and the metal ion stock solutions in PCR tubes (strips of 8 tubes). After pipetting 1 μL of the stock solution, wash the tip once by pipetting up and down with 1.5 μL within the sample. Change the pipette tips and mix by pipetting up and down three to five times with a volume of 50 μL. We recommend using electronic multichannel pipettes that have a programmable “pipette and mix” option. Avoid air bubbles while mixing. Pipetting mistakes can be identified by inspection of the signal after addition of the positive control ionophore and comparing it to the expected value. For technical reasons, plate readers may be localized in climatized rooms with temperatures set close to 20 °C. Since these temperatures might be close to the phase transition of the used lipid mixture, it is possible that the measurements will be compromised if the sample is not measured fast enough. Although the integrity of the proteoliposomes and the quality of the measurement is controlled by the final addition of Calcimycin or CCCP, we advise the use of a heating block set to 22 °C to store the proteoliposome stock to be measured. As high concentrations of divalent ions influence liposome stability and might trigger liposome fusion, it is important to apply the described positive and negative control experiments.

*Notes:*

*1. The timing of the sequence may vary depending on the purpose of the experiment and transport properties. The sequence outlined above is suitable for the investigation of metal ion transporters studied in the Dutzler laboratory. To monitor proton transport, steps D3b–c were performed in reverse order, as adding valinomycin before the substrate led to a significant decrease in fluorescence due to the uncoupled proton leak.*

*2. Cuvette-based fluorimeters could provide higher sensitivity and allow reagent addition during measurement, which can be an advantage for experiments requiring higher temporal resolution. However, measurements in a cuvette require larger sample volumes compared to plate-based assays, which may limit their practicality when working with purified membrane proteins, where protein yield is often limited.*

**Figure 3.**
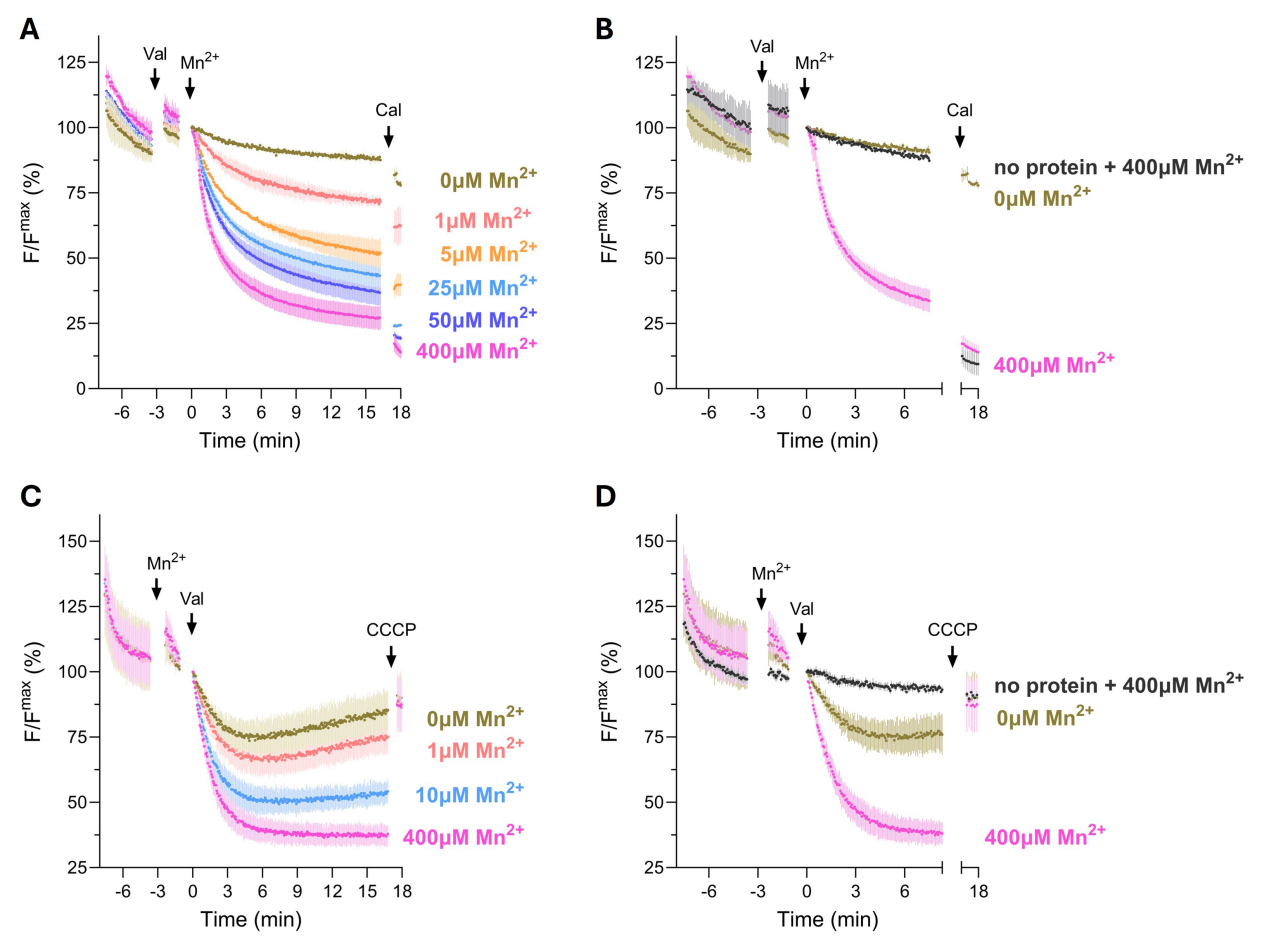
Transport properties of the prokaryotic divalent metal transporter EcoDMT. (A) Mn^2+^ transport into EcoDMT proteoliposomes containing EcoDMT, assayed by the quenching of the fluorophore Calcein trapped inside the vesicles. (B) Mn^2+^ transport, including the control of empty liposomes containing no protein, and in the presence of the highest Mn^2+^ concentration used in the assay. For comparison, the protein data without Mn^2+^ and with 400 μM Mn^2+^ are displayed. (C) Mn^2+^ coupled H^+^ transport into EcoDMT proteoliposomes assayed by the quenching of the fluorophore ACMA. (D) Mn^2+^ coupled H^+^ transport, including the control of empty liposomes containing no protein, and in the presence of the highest Mn^2+^ concentration used in the assay. For comparison, the protein data without Mn^2+^ and with 400 μM Mn^2+^ are displayed. After equilibration of the fluorescent signal, the K^+^ ionophore valinomycin (Val) and the substrate Mn^2+^ were added at the indicated time points to establish a negative membrane potential and to start the transport. At the end, the Mn^2+^ ionophore Calcimycin (A) or H^+^ ionophore CCCP (B) was added as an internal positive control. In all cases, the mean of three experiments from three independent experiments is displayed. The data is normalized to the value after the addition of substrate or valinomycin (t = 0). Applied micromolar ion concentrations of Mn^2+^ to the outside are indicated on the right. A subset of these data is published in [14] in Figures 2A and 4H.

4. Perform a linear regression in the linear range of signal change to obtain initial transport velocities. In our assays, the linear range usually comprised data of approximately 0.7 min of measurement, 1 min after starting the transport. Analyze these values with respect to the substrate concentration using a Michaelis–Menten equation to obtain K_M_ and V_max_ values.

5. Normalize the data to the fluorescence signal at the time point of substrate addition and plot the data. When using Fura-2, normalization is done by calculating the ratio F_340_/F_380._ Typical data is shown in Figure 3.

## Validation of protocol

For each experimental condition, at least two independent proteoliposome reconstitutions have been analyzed, and at least three independent biological replicates were performed. The respective number of replicates is stated in the figure legends of the research articles listed below. Positive controls included known metal ion transporters with well characterized transport properties (EcoDMT in most cases, Figure 3A and C) and the addition of ionophores such as Calcimycin, Ionomycin, or CCCP to assess maximum fluorescence signals. Negative controls consisted of protein-free liposomes (data displayed in black in Figure 3B and D). Further validation of the assay can be provided by unrelated membrane proteins, inactive transporters due to point mutations, or specific inhibitors. This supports the conclusion that the observed signal arises from protein-mediated transport rather than passive leakage driven by the chemical gradient or membrane potential.

This protocol (or parts of it) has been used and validated in the following research articles:

- Ehrnstorfer et al. [36], DOI: 10.1038/nsmb.2904, Crystal structure of a SLC11 (NRAMP) transporter reveals the basis for transition-metal ion transport, *Nat Struct Mol Biol*, 2014 (Figure 1b, c, Figure 2k, I, Supplementary Figure 6).
- Ehrnstorfer et al. [9], DOI: 10.1038/ncomms14033, Structural and mechanistic basis of proton-coupled metal ion transport in the SLC11/NRAMP family, *Nat Commun*, 2017 (Figure 1, Figure 4c, c, Figure 6, Supplementary Figure 1b–e).
- Manatschal et al. [12], DOI: 10.7554/eLife.51913, Mechanistic basis of the inhibition of SLC11/NRAMP-mediated metal ion transport by bis-isothiourea substituted compounds, *eLife*, 2019 (Figure 2a, Figure 2 – figure supplement 1, Figure 4 – figure supplement 2).
- Ramanadane et al. [14], DOI: 10.7554/eLife.74589, Structural and functional properties of a magnesium transporter of the SLC11/NRAMP family, *eLife*, 2022 (Figure 2, Figure 2 – figure supplement 1, Figure 2a–f, Figure 4, Figure 8b–i).
- Lehmann et al. [10], DOI: 10.7554/eLife.83053, Structures of ferroportin in complex with its specific inhibitor vamifeport, *eLife*, 2023 (Figure 1a, Figure 1 – figure supplement 1c).
- Ramanadane et al. [13], DOI: 10.7554/eLife.85641, Structural and functional properties of a plant NRAMP-related aluminum transporter, *eLife*, 2023 (Figure 1a–g, j–l, Figure 1 – figure supplement 2c–k, Figure 5b–e, g–k).
- Liziczai et al. [11], DOI: 10.1038/s41467-024-54705-0, Structural basis for metal ion transport by the human SLC11 proteins DMT1 and NRAMP1, *Nat Commun*, 2025 (Figure 1, Figure 2, Figure 6b–e, Supplementary Figure 1, Supplementary Figure 2, Supplementary Figure 11, Supplementary Table 1, Supplementary Table 2).

## General notes and troubleshooting

**General notes**

In general, the assay is highly sensitive to numerous variables (e.g., temperature, timing between extrusion and measurement, and other small procedural differences). Therefore, it is essential to always include appropriate positive and negative controls, such as an established transporter or wild-type protein, and protein-free liposomes, to ensure reliable interpretation of results (example data are displayed in Figure 3). In our assay, the negative control was prepared in parallel to the reconstitution of the protein of interest using the exact same protocol, with the difference that a size exclusion chromatography buffer devoid of protein was used [14,28]. This approach allows monitoring of complete detergent removal, which is critical for ensuring liposome tightness. Incomplete detergent removal during liposome preparation can lead to leaky vesicles, even in the absence of protein. Thus, verifying the integrity of protein-free liposomes is essential to confirm that any observed transport activity is indeed protein-mediated.

It is advisable to perform all experimental steps on the same day, as unilamellar proteoliposomes are only stable for a limited time before multilamellar structures begin to form. As the fluorescent lamp varies between instruments, we advise adjusting the Tecan gain for each fluorophore at the aforementioned concentration and keeping it the same for all measurements.

**Troubleshooting**

**Problem 1:** Fluorescence level remains rather high even after adding the positive control (Calcimycin, Ionomycin, or CCCP).

Possible cause: Presence of multilamellar liposomes.

Solutions: Perform a longer extrusion to ensure unilamellarity (generally, 17–25 cycles), minimize the time between extrusion and the start of the measurement, and keep the temperature above the phase transition temperature of the lipid mixture. In case of liposome preparation by sonication, repeat the sonication step.

**Problem 2:** Noisy baseline or increased noise after the addition of ionophore or metal ion solution.

Possible cause: Presence of an air bubble in the measurement well.

Solutions: Gently tap the plate on the table to dislodge bubbles before starting the measurement or carefully remove bubbles using a fine needle.

**Problem 3:** Significant signal in the negative control (empty liposomes).

Possible cause: Insufficient removal of detergent.

Solutions: Detergents with very low critical micelle concentrations (CMC) may be more difficult to remove during reconstitution using biobeads. In such cases, increasing the amount of biobeads or extending the incubation time can improve detergent removal.

**Problem 4:** Low transport signal in transport assay.

Possible cause: Insufficient reconstitution efficiency or low activity of the protein.

Solutions: The optimal protein-to-lipid ratio depends on the activity of the transporter and requires empirical optimization. Reconstitution efficiency can be assessed by re-extracting the protein from proteoliposomes and quantifying the protein amount using appropriate methods (e.g., SDS-PAGE or chromatography). In case of persistent difficulties of reaching a good reconstitution efficiency, alternative methods should be tested, such as rapid dilution, dialysis, or size exclusion chromatography (example protocols can be found in [33–35] and supplements of [27].
